# THER: Integrative Web Tool for Tumour Hypoxia Exploration and Research

**DOI:** 10.1111/cpr.70053

**Published:** 2025-05-01

**Authors:** Yasi Zhang, Huiying Liu, Pengpeng Zhang, BiCheng Ye, Haoxuan Ying, Hong Yang, Jian Zhang, Nan Zhang, Kailai Li, Ting Wei, Aimin Jiang, Anqi Lin, Peng Luo

**Affiliations:** ^1^ Donghai County People's Hospital Jiangnan University Smart Healthcare Joint Laboratory, Donghai County People's Hospital (Affiliated Kangda College of Nanjing Medical University) Lianyungang China; ^2^ Department of Oncology Zhujiang Hospital, Southern Medical University Guangzhou China; ^3^ Department of Lung Cancer, Tianjin Lung Cancer Center, National Clinical Research Center for Cancer, Key Laboratory of Cancer Prevention and Therapy, Tianjin's Clinical Research Center for Cancer Tianjin Medical University Cancer Institute and Hospital Tianjin China; ^4^ Liver Disease Center of Integrated Traditional Chinese and Western Medicine, Department of Radiology Zhongda Hospital, Medical School, Southeast University, Nurturing Center of Jiangsu Province for State Laboratory of AI Imaging & Interventional Radiology (Southeast University) Nanjing China; ^5^ College of Life Science and Technology Huazhong University of Science and Technology Hongshan China; ^6^ Department of Urology, Changhai Hospital, Naval Medical University Second Military Medical University Shanghai China

## Abstract

THER: Integrative Web Tool for Tumor Hypoxia Exploration and Research.

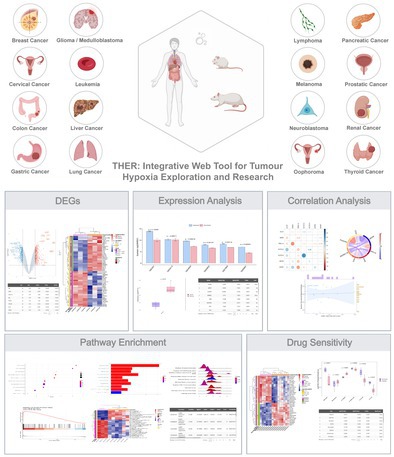

Dear Editor,

Tumour hypoxia refers to the gradual decrease in the rate of ATP production in cells or tissues when the partial pressure of oxygen drops below a critical level, and hypoxia occurs within the tumour, thus contributing to the malignant development of tumour cells to adapt to their environment [1]. Several studies have shown that a series of changes induced by hypoxia play an indispensable role in tumour development [2]. Under the selective pressure of hypoxia, tumours can make themselves invasive or metastatic through various mechanisms [3, 4]. Furthermore, based on the importance of hypoxia in tumorigenesis and development, hypoxia phenotypes have been used in several studies to explore their importance in tumour prognosis [5, 6]. However, the effect of hypoxia on the development, progression and sensitivity to drugs may be quite different in different tumours [7, 8] but the mechanistic differences have not yet been clarified. Therefore, we hope to explore the mechanisms of hypoxia‐induced tumorigenesis and development, identify new markers for assessing hypoxia‐induced tumorigenesis and development, and also identify effective drugs for treating hypoxic tumours.

There is an extremely rich amount of transcriptomic data stored in public databases, providing an unprecedented possibility to study the effect of hypoxia on tumorigenesis, progression and drug resistance. However, analysis of transcriptomic data to explore the link between hypoxia and the mechanisms of tumorigenesis and progression still requires a certain programming foundation, which poses a great challenge for most clinical researchers who do not have basic programming knowledge.

Therefore, we developed an online web analysis tool called THER. On this platform, users can analyse the results of their research based on 63 curated tumour hypoxia‐associated transcriptomic data sets (Table S1) from the Gene Expression Omnibus database, and based on different oxygen states (hypoxia/normoxia), differential expression, expression profiling, correlation, enrichment and drug sensitivity analyses can be easily and quickly performed. THER is open to all users and can be accessed without login at https://smuonco.shinyapps.io/THER/.

The foundation of THER's analytical power lies in its rigorously curated data sets. We searched the Gene Expression Omnibus database with the keyword “hypoxia” and filtered results. We only included (i) tumour data sets, (ii) samples treated with hypoxia or normoxia only, (iii) data sets with more than three samples in both the hypoxia and normoxia groups, (iv) data for *Homo sapiens, Mus musculus or Rattus norvegicus* and (v) transcriptomic data sets.

For data preprocessing, methods varied by data source. RNA‐seq used DESeq [9] for raw count processing including low‐abundance filtering, differential analysis and normalisation; Affymetrix arrays applied affy [10] (HGU95/HGU133/MGU74: ReadAffy with rma background correction and normalisation) or oligo [11] (other platforms) with equivalent procedures; Illumina BeadArray employed lumi [12] (lumiR.batch for background correction and log2 transformation followed by lumiExpresso quantile normalisation); Agilent arrays utilised backgroundCorrect and normalizeBetweenArrays functions of limma [13].

Having completed the data preprocessing through the diverse methods tailored to each data source, we moved forward to develop the THER platform. This platform contains five essential analysis modules, namely, the differential expression analysis module, expression profiling module, correlation analysis module, enrichment analysis module and drug‐sensitivity analysis module (Figure 1). Users can use these analysis modules to autonomously explore various features in tumour samples under hypoxia/normoxia conditions to investigate the changes that occur in tumours in hypoxic environments. Users are free to select the data set of interest and can choose to display custom genes/pathways/drugs or visualise the top genes/pathways/drugs in descending order by |log2FC| value.

**FIGURE 1:**
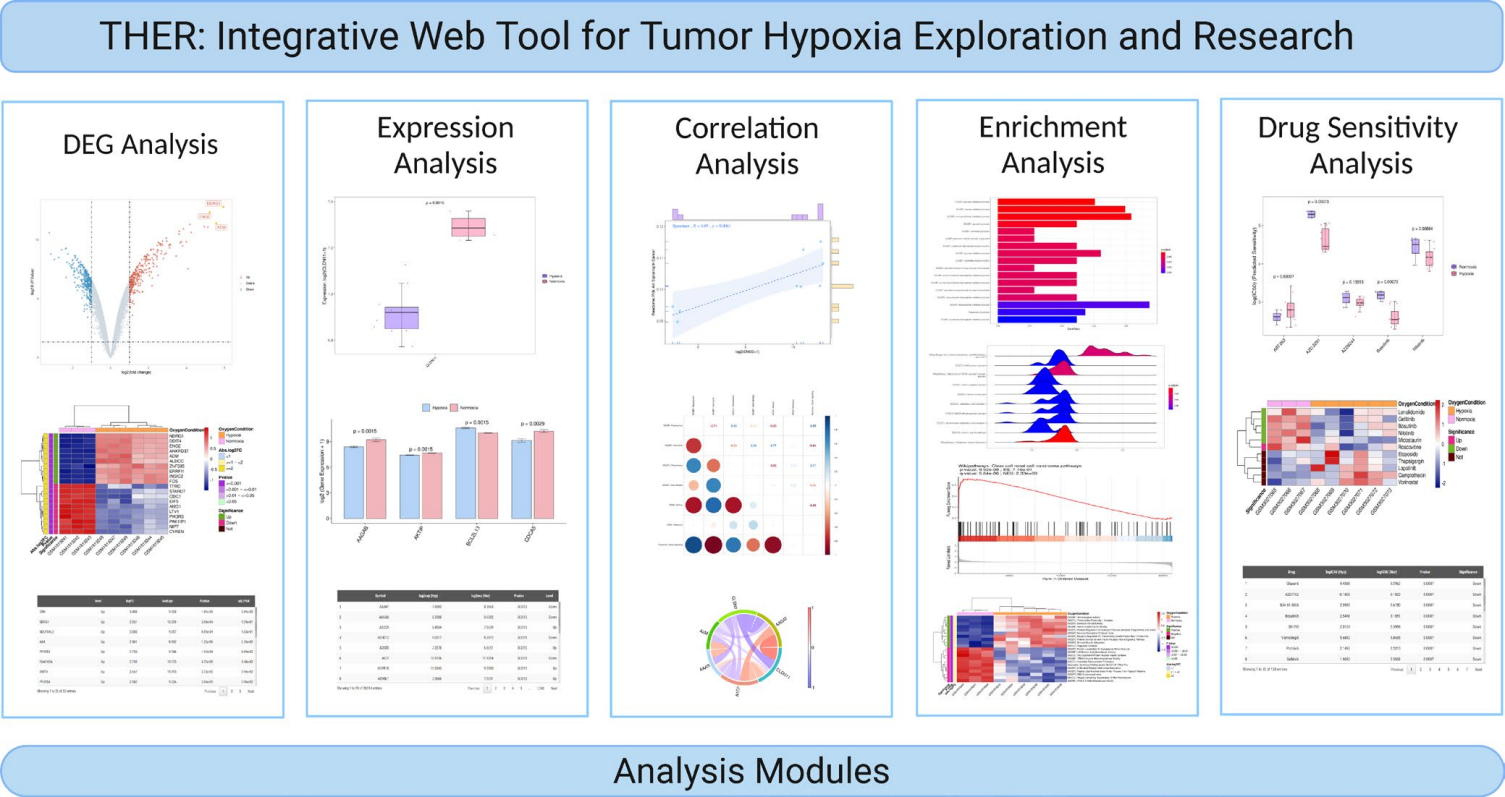
Analysis modules of THER.

Below the Data module, general information about the 63 data sets included in THER is shown, and more detailed information about the data sets is available to the user by clicking on the plus button on the left side of each column in the table; thus, the user can quickly locate the data set of interest and jump directly to the analysis module of the corresponding data set by clicking on the black jump button in the first column of the table.

The DEG analysis module consists of three sub‐modules, volcano, heatmap and table (limma), which allow the user to compare the differences in gene expression between the normoxia and hypoxia groups and to present the results of the limma differential expression analysis according to oxygen status in different formats. Specifically, the sub‐module volcano allows the user to visualise volcano plots indicating whether the expressions of genes are significantly up‐regulated, significantly down‐regulated or not significantly regulated in the hypoxia group relative to the normoxia group for highlighting statistically significant changes. The sub‐module heatmap allows users to visualise heatmaps illustrating the expression levels of genes in different samples and the results of significant differences in different oxygen states for an intuitive visualisation of gene expression patterns. The table sub‐module allows users to view the results of the differential expression analysis in tabular formats, including the level of significant difference, |log2FC| value, mean expression value or count mean, *p* value and adjusted *p* value for detailed numerical comparisons.

The expression module consists of three sub‐modules, boxplot, barplot and table (wilcox‐test), which allow users to compare the expression differences of single or multiple genes between the normoxia and hypoxia groups, to present the expression values of the genes in different oxygen states in different formats and to determine whether the genes being investigated are significantly differentially expressed genes between oxygen states by using a Mann–Whitney U test. The Mann–Whitney U test was used to determine whether the genes explored were significantly differentially expressed genes between oxygen states.

The correlation analysis module contains three sub‐modules, scatter, correlation heatmap and chord diagram, which allow the user to freely explore the correlation between the expression of different genes, the expression of genes and the relative expression activity of pathways, or the relative expression activity of different pathways, and present the visualisation results in the form of scatter plots, correlation heatmaps or chord diagrams.

The Enrichment Analysis module consists of five sub‐modules: dotplot/barplot, ridgeplot, GSEA plot, heatmap (GSVA) and table, which present the results of differentially enriched pathways in different formats. This module helps users correlate genes with functions and provides an opportunity to discover potential biological pathways related to tumour hypoxia.

The drug‐sensitivity analysis module contains three sub‐modules which are designed to provide insights into the differential responses of various samples to therapeutic drugs under differing oxygen conditions. The three sub‐modules present the results of the drug‐sensitivity analyses in different formats and use the Mann–Whitney U test to determine whether or not the differences in the log‐transformed half‐maximal inhibitory concentration (IC50) values for the drugs presented are significant between samples in the hypoxia and normoxia groups.

To verify the reliability of our web tools, we conducted an experimental verification (Figure 2). Through the drug‐sensitivity analysis module in the web tool we developed, we observed significant differences in chemotherapeutic drug sensitivity between different tumour cell lines under hypoxic and normoxic conditions. For example, the IC50 values of the hypoxic‐treated MCF7 cell line for multiple chemotherapeutic drugs showed significant differences, which suggests that hypoxic environments significantly modify the sensitivity of MCF7 cells to chemotherapeutic drugs. Then, we further extended our study with the web tool CPADS [14] and observed that lung cancer affects resistance to commonly used chemotherapeutic agents through the hypoxia pathway. To thoroughly substantiate our web‐based tool findings, we conducted experimental verification using three lung cancer cell lines and five drugs (Table S2). The results confirmed that there were significant differences in the IC50 values of tumour cells cultured under hypoxic conditions for specific chemotherapeutic drugs, indicating that hypoxic conditions significantly affected the sensitivity of tumour cells to drugs. This indicates that hypoxia affects drug efficacy against tumours across various histological subtypes and drug classes. The consistency between the web tool predictions and experimental findings underscores the robustness and reliability of our web tool, bolstering confidence in its applicability to both research and clinical contexts.

**FIGURE 2:**
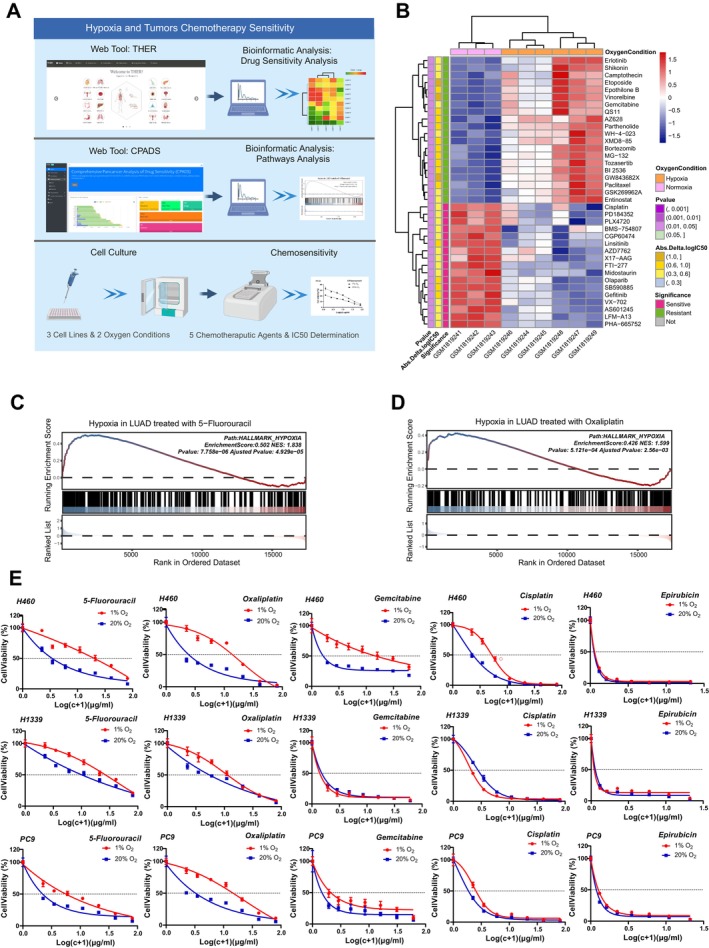
The differences in chemotherapeutic sensitivity of tumour cell lines under hypoxic and normoxic conditions. (A) Differences in chemotherapeutic drug sensitivity of tumour cell lines under different oxygen conditions were found by the web tool THER, followed by the web tool CPADS to verify that hypoxia may affect the chemotherapeutic sensitivity of tumour cells. Subsequently, we cultured three tumour cell lines under different oxygen conditions and determined their sensitivity to five common chemotherapeutic drugs. (B) Differences in IC50 values and sensitivities of MCF7 cell lines to multiple chemotherapeutic drugs under hypoxic and normoxic conditions in the GSE29406 data set. (C, D) Lung cancer affects resistance to 5‐fluorouracil and oxaliplatin through the HALLMARK_HYPOXIA pathway. (E) Differential sensitivity to 5‐fluorouracil, oxaliplatin, epirubicin, gemcitabine and cisplatin under hypoxic and normoxic conditions in H460, PC9 and H1339 cell lines.

In summary, hypoxia plays a crucial role in tumorigenesis and in enhancing tumour invasiveness, necessitating the clarification of its molecular mechanisms and the identification of related drugs and markers. Currently, there are no other web‐based tools dedicated to comprehensively studying the mechanisms of hypoxia on tumour development. To this end, we developed THER, an online web tool. It contains 63 transcriptome data sets from the GEO database, covering 3 species, 18 tumour types, and 42 cell line types, along with five analysis modules for biomarker screening, mechanism exploration and drug‐sensitivity analysis. THER holds value in clinical translation. It helps non‐programming‐skilled clinicians and researchers identify potential targets and drugs for hypoxic tumours. Biomarker screening using THER can assist doctors in identifying potential diagnostic markers, thus improving diagnostic efficiency. The drug‐sensitivity module can guide clinicians to select more effective drugs for patients, enhancing treatment efficiency.

While THER provides novel functionalities, users should be aware that technical variability across source data sets could introduce batch effects [15] during cross‐cohort analyses. Moreover, hypoxia‐driven genes and pathways often exhibit substantial variability depending on tumour microenvironment, genetic background and tissue‐specific regulatory mechanisms. This heterogeneity complicates the generalisability of biomarkers or therapeutic targets identified from single cancer types, necessitating cautious interpretation of pan‐cancer analyses. To expand THER's scope, integrating epigenomic data like DNA methylation profiles will reveal epigenetic changes in tumours during hypoxia. Proteomic data integration can identify hypoxia‐related proteins, which may serve as biomarkers or therapeutic targets, strengthening THER's capabilities.

## Author Contributions

Yasi Zhang is responsible for conceptualisation, methodology, formal analysis, data organisation, visualisation, initial draft writing and editing. Huiying Liu is responsible for investigation, resources, data curation, data organisation and review. Pengpeng Zhang, BiCheng Ye and Haoxuan Ying are responsible for conceptualisation, methodology, validation, investigation, review, editing and project management. Hong Yang, Jian Zhang, Nan Zhang and Kailai Li are responsible for conceptualisation, methodology, validation, review, editing and supervision. Wei Ting, Aimin Jiang, Anqi Lin and Peng Luo are responsible for conceptualisation, validation, review, supervision, project management, resource and funding acquisition and editing.

## Ethics Statement

The authors have nothing to report.

## Conflicts of Interest

The authors declare no conflicts of interest.

## Supporting information


**Table S1.** Comprehensive Information of 63 Tumour Data sets: Hypoxia Treatment Groups and Normoxia Control Groups.

**Table S2.** Cell viability of 3 cell lines treated with various concentrations of 5 chemotherapeutic drugs under 1% and 20% oxygen conditions.

## Data Availability

The data that support the findings of this study are openly available in Gene Expression Omnibus at https://www.ncbi.nlm.nih.gov/geo/, reference number GSE69833 et al.
